# Set aside—A qualitative study of partners’ experiences of pregnancy, labour, and postnatal care in Sweden during the COVID-19 pandemic

**DOI:** 10.1371/journal.pone.0307208

**Published:** 2024-09-06

**Authors:** Sophia Holmlund, Karolina Linden, Anna Wessberg, Verena Sengpiel, Cornelia Appelgren, Lisa Lundmark, Maria Lindqvist

**Affiliations:** 1 Department of Nursing, Umeå University, Umeå, Sweden; 2 Department of Clinical Sciences, Obstetrics and Gynecology, Umeå University, Umeå, Sweden; 3 Judith Lumley Centre, School of Nursing and Midwifery, La Trobe University, Bundoora, Victoria, Australia; 4 Institute of Health and Care Sciences, Sahlgrenska Academy, University of Gothenburg, Gothenburg, Sweden; 5 Department of Obstetrics and Gynecology, Region Västra Goötaland, Sahlgrenska University Hospital, Gothenburg, Sweden; 6 Department of Obstetrics and Gynaecology, Institute of Clinical Sciences, Sahlgrenska Academy, University of Gothenburg, Gothenburg, Sweden; De Montfort University, UNITED KINGDOM OF GREAT BRITAIN AND NORTHERN IRELAND

## Abstract

**Background:**

Due to changes in Swedish maternity care during the COVID-19 pandemic, partners were often excluded from antenatal and postnatal care.

**Aim:**

To explore partners’ experiences of pregnancy, labour, and postnatal care in relation to the COVID-19 pandemic restrictions.

**Methods:**

A descriptive qualitative interview study with 15 partners of women who gave birth from March 2020 to March 2022. Data was collected from April to November 2022, and analysed using inductive thematic analysis.

**Findings:**

Two themes and six subthemes were identified. The first theme, *Feelings of loss and exclusion*, emphasises the expectation and desire to share the journey of becoming a parent together with the pregnant partner. When excluded from maternity care, a feeling of missing out was described which could create a sense of distance from the unborn child. The second theme, *Dealing with powerlessness*, relates to the fear of infection and not being able to participate during the birth, and life being adapted to restrictions. Mixed feelings regarding the restrictions were described since the reasons behind were not always perceived as clear and logical.

**Discussion:**

Sweden prides itself on gender equality, where partners normally are a natural part of maternity care. This likely contributed to strong feelings of exclusion when partners were prevented from participating in maternity care during the COVID-19 pandemic.

**Conclusion:**

Partners of women giving birth during the COVID-19 pandemic were substantially affected by the restrictions within maternity care. Partners wish to be involved in pregnancy and birth and want to receive clear information as part of their preparation for parenthood. Society–including maternity care–must decide how to address these needs.

## Introduction

The COVID-19 pandemic started to affect Sweden during the early Spring of 2020 [1]. International studies reported that pregnant women with COVID-19 had an increased risk of admission to intensive care, neonatal death, preterm birth and neonatal care for their infants [2]. Health-related worries rose significantly in pregnant women and their partners as a consequence of the uncertainties connected to the pandemic [3]. At the same time, healthcare workers caring for these women experienced difficult working conditions, with long hours, lack of recovery, and fear of becoming infected [4]. To counteract the spread of the virus and ensure a functioning healthcare system, service provisions were adapted, and maternity care (antenatal-, intrapartum- and postnatal care) became focused solely on the pregnant woman and her foetus. Antenatal care was offered in person, and the partners of pregnant women were generally not allowed to accompany their partners to appointments. In 2020, all healthcare regions in Sweden had restrictions for partners’ participation in antenatal care and/or ultrasound examinations [5]. This led to women feeling isolated and lacking support during pregnancy, especially when pregnancy complications arose [6]. Healthy partners without symptoms of COVID-19 were usually allowed to attend the birth of their children, but different healthcare regions had different approaches to whether the partner could remain in the postnatal ward after the baby had been born [6]. Swedish COVID-19 vaccination administration started in January 2021 [7], and was available for pregnant women with risk factors from April 2021 [8]. Sweden did not go into lockdown, and instead adopted a strategy of strict recommendations in order to reduce the spread of COVID-19. These recommendations included social distancing, working from home if possible, and economic compensation for those most vulnerable to severe COVID-19 to stay home from work [9]. Restrictions on social gatherings were put in place, affecting parental education classes, baby activities, and private social gatherings [10]. Most of the measures put in place to prevent the spread of COVID-19 in Sweden were lifted in February 2022 [8].

Childbirth can be a shared experience for the couple, with them preparing, managing labour, and making decisions regarding the birth together [11]. Swedish fathers described lacking care support and missing out on meeting other fathers-to-be during the COVID-19 pandemic [12]. This was mirrored in the UK, where father exclusion in maternity care led to feelings of isolation and a sense of loss of and disconnection from the pregnancy [13]. Norwegian co-parents have also described that exclusion from maternity care can result in stress, insecurity, worry, and shame [14]. The experience of ‘missing out’ appears to be consistent between countries, and partners experienced increased psychological stress during pregnancy, labour, and the postnatal period due to not being part of the process or involved in decision-making [13, 15, 16]. Further, fathers who were not allowed to participate in the birth of their child described feelings of distress and a deferred transition to parenthood [17].

Experiences of pregnancy for non-birthing parents are influenced by the surrounding context [18]. In Sweden, the father or non-birthing partner is widely recognised as naturally having an active role in pregnancy, labour, and parenthood [19]. Due to the restrictions during the COVID-19 pandemic, the involvement of partners was challenged. Thus, the aim of this study is to explore partners’ experiences of pregnancy, labour, and postnatal care in relation to the COVID-19 pandemic.

## Methods

### Design

Between April and November 2022, a descriptive qualitative study with inductive thematic analysis [20] of semi-structured interviews was conducted. This research design is particularly useful when there is limited knowledge about the area of study, and the analysis remains closely tied to the data [21]. The findings are reported according to the Standards for Reporting Qualitative Research (SRQR) checklist [22].

This study is part of a larger research project called COPE (Covid-19 in Pregnancy and Early Childhood, NCT04433364), which is a national, multi-centre study of the impact of the COVID-19 pandemic on pregnant women, their babies and partners [23].

### Study context

The study was conducted in two areas in Sweden; one in the southwest, which is one of the three most populated regions in Sweden, and the other in the north, with a low population density [24]. All of the infants of the participants were born at university hospitals. In order to contextualise the study, the Swedish maternity care system is described in Table 1 [19, 25–27].

**Table 1:** Maternity care* in Sweden.

Key aspects of the Swedish maternity care system
• Free maternal health service with an almost universal uptake among pregnant women.
• Generally, antenatal care is provided as part of public healthcare services. Both public and private care are government-funded.
• Most antenatal care clinics operate within primary care, but can also be managed by hospitals. Women often attend the antenatal care clinic closest to their place of living, but can choose another clinic if they prefer.
• Antenatal care is organised in maternal healthcare areas. Each area has an antenatal care coordinator (midwife) and an antenatal care obstetrician; responsible for improving and evaluating antenatal care as well as providing local guidelines and education to midwives and physicians.
• Midwives in Sweden initially train as nurses at Bachelor’s level, then spend 18 months studying midwifery at an advanced level. This gives a total of 4.5 years of university studies.
• Midwives are responsible for normal pregnancy, labour, and postnatal care; obstetricians become involved only in the event of complications.
• Most pregnant women meet the same midwife or few midwives throughout pregnancy and/or the routine postpartum follow-up visit; however, continuity of carer throughout pregnancy, labour, and postpartum is uncommon.
• A detailed programme, including content of each antenatal care visit, is provided by regional healthcare services and based on national recommendations.
• A minimum of eight visits to antenatal care are recommended during pregnancy, and an additional visit is recommended eight to twelve weeks postpartum.
• All pregnant women are offered at least one ultrasound examination, with local differences in provision of other prenatal diagnostic methods. These methods are also provided by private practitioners, generally located in urban areas.
• Midwives in antenatal care are obliged to provide information and support to expecting parents, in addition to medical assessments of pregnancy.
• Partners are normally involved in pregnancy, labour, and postnatal care.
• Labour, birth, and postnatal care are largely provided in a traditional model at hospitals. Home births are rare.
• Postnatal care is provided at the hospital for a few days (mean 1.6 days after vaginal birth and 2.7 days after caesarean section), or through follow-up visits at the hospital or at home by a midwife after early discharge (>six hours).
• Specially trained nurses at the child healthcare centres provide follow-up visits from approximately one week after birth.

* Antenatal-, intrapartum- and postnatal care

### Participants

Participants were recruited using purposive sampling, aiming for a broad variation of participants [28]. The inclusion criteria were partners of women who had been pregnant and given birth in Sweden during the COVID-19 pandemic, and were speaking Swedish or English to be able to take part in an interview. All of the participants lived with the other parent. Births occurred between March 2020 and March 2022. The sociodemographic variables of the participants (n = 15) are presented in Table 2.

**Table 2:** Sociodemographic characteristics of the participants.

Variables	
***Participants (n)***	15
***Age (years)***	
Mean	34
Min–Max	26–41
***Gender***	
Male	14
Female	1
***Highest level of education***	
University	10
Upper-secondary education (ages 16–19)	5
***Area of Sweden***	
The northern area	6
The southwestern area	9
***Country of origin***	
Sweden	12
Europe (other than Sweden)	2
Outside Europe	1
***Parenthood***	
First-time parent	10
Second-time parent	5
**Year of birth of the baby**	
2020 (March-December)	8
2021 (January-December)	5
2022 (January-March)	2

### Data collection

Information about the study was first posted on an Instagram page belonging to a birth unit in the southwest area with 34,900 followers. This post invited eligible partners to contact the research team via email for more information about the study and to schedule a time for the interview. During the process of conducting the interviews, it emerged that most participants had an academic degree and were living in the same area. In order to have a broader range of sociodemographic characteristics, the authors recruited participants through antenatal care services in the northern area, aiming for partners without university education. Midwives were asked to identify and provide information regarding the study to partners who met the criteria, then pass on the contact information of interested partners to the authors, leading to an additional three recruited partners. Furthermore, the researchers possessed the contact information of women and partners who had previously consented to participation in other parts of the COPE study [23]. By contacting these partners, another two participants were recruited.

A semi-structured interview guide was constructed, which included one main question “Can you tell me about your experience of having a child during the COVID-19 pandemic?” in relation to the domains of pregnancy, labour, and the postnatal period. Follow-up questions such as “How did you experience that?” and “Can you tell me more about that?” were posed. Most of the interviews were conducted by SH (9 of 15), with additional interviews by CA, LL, and AW. Two of the interviewers, SH and AW, are midwives and researchers with experience of performing interviews. CA and LL were midwifery students trained by SH before moderating their own interviews. The interviews were conducted via digital video meetings and lasted 28–65 minutes (mean: 44 minutes). The last interviews repeatedly revealed similar aspects of the study area, leading to the conclusion that 15 interviews were sufficient. The interviews were audio recorded and transcribed verbatim in Swedish by a professional transcription company (n = 10) and by researchers CA and LL (n = 5).

### Analysis

A manual thematic analysis with an inductive approach was performed, according to Braun and Clarke [20]. This method includes six steps to identify themes or patterns in qualitative data [20].

By reading and re-reading the transcribed material, the authors became familiar with the data and able to produce initial codes. Two of the authors (CA and LL) coded half of the material each, with double coding by SH. The authors coded for as many themes as possible in order to ensure that nothing was missed. The codes were compiled into a list and sorted into potential themes. All codes not meeting the study aim were removed. When all data had been coded, CA, LL, SH and ML discussed the codes together and sorted them into themes and subthemes co-ordinately. The themes were reviewed and refined until consensus was achieved. The transcribed interviews were then reread to make sure that the themes were coherent with the data, and themes were named. In the last step, the analysis was discussed and confirmed by KL, AW and VS, and the report was produced. The quotes were translated from Swedish to English by SH and accuracy was checked by KL, AW, VS and ML. An example of the data analysis process is presented in Table 3.

**Table 3:** Examples of the data analysis process.

Data extract	Code	Subtheme	Theme
In retrospect, I’m quite disappointed at how badly they adjusted. . . yes, mainly on antenatal care, you could say… . Yes, but it is probably the fact that we were not offered any alternatives, that you were completely excluded from. . . No meeting was digital.	Lack of digital alternatives created feelings of exclusion in antenatal care	Facing a changeable maternity care	Feelings of loss and exclusion
If it was a caesarean section, should she be lying there and not be able to get up herself and take care of the baby? And what kind of resources do they have if there is no partner there? It created a huge amount of stress before, I must say.	Huge stress stems from uncertainty about the staff’s resources to replace the partner’s support	A constantly present worrying mind	Dealing with powerlessness

In terms of personal reflexivity, some of the authors (KL, AW, VS) have a broad pre-understanding and interest of the area under study as this is part of a broader research project on COVID-19. All of the authors have a clinical background in midwifery or obstetrics, with AW, VS, CA and LL working in maternity care during the COVID-19 pandemic. Seeing the challenges faced by pregnant women, their partners, and healthcare professionals due to the virus and restrictions may influence the results.

### Ethics

Ethics approval was granted for this study by the Swedish Ethical Review Authority (diary number 2020–02189, May 12^th^ 2020, amendments 2020–02848, June 4^th^ 2020, 2020–05016, September 28^th^ 2020, 2022-01231-02, March 9^th^ 2022) before the interviews were conducted. Eligible participants received oral and written information about the study, and were informed that participation was voluntary. Informed written consent was obtained before the start of each interview. To ensure confidentiality, all results are presented on a group level, and quotations are assigned an interview number.

## Results

The thematic analysis yielded two themes and six subthemes (Table 4).

**Table 4:** Overview of themes and subthemes.

Theme	Subtheme
**Feelings of loss and exclusion**	Facing a changeable maternity care
	Reflecting upon the effects of disconnection
	Striving to provide support
**Dealing with powerlessness**	A constantly present worrying mind
	Alternating between accepting and struggling with incomprehensible restrictions
	Adjusting life to minimise the risk of infection

### Feelings of loss and exclusion

The first theme identified was *Feelings of loss and exclusion*, which related to the practical and emotional effects of not being involved as a partner in maternity care. The participants had an expectation and a need to share the journey of becoming a parent with their pregnant partner. The participants valued the antenatal care appointments, and when they were shut out of these a feeling of missing out was described. However, this was less frequent for those becoming a parent at the end of the pandemic. Receiving support from midwives and being able to provide support to their partner throughout pregnancy, labour, and the postnatal period was described as important. Exclusion from maternity care could create a sense of loss of involvement in parenthood.

#### Facing a changeable maternity care

The participants stated that the restrictions in maternity care varied over time, leading to a changing impact on their lives. All of the participants were allowed in the labour room despite the restrictions, but not all were allowed in the postnatal ward. The antenatal care appointments were affected in different ways due to restrictions; some participants were allowed to attend throughout the pregnancy, but the majority were not allowed to participate in any or most of these appointments including ultrasound appointments.

*´It was only approximately halfway through the pregnancy that the restrictions came into force. And I wasn’t allowed to attend the antenatal care appointments anymore. And there would be no parental education classes in groups, and no preparing… And we had kind of looked forward to attending those.´* (Interview 7)

A few reported that they did not feel that they had missed out by not being able to attend the antenatal care appointments, expressing an understanding that antenatal care mainly revolves around the person who is pregnant and the foetus.

*´It wasn’t that big of a deal for me, because it felt like those questions and the things they were doing there maybe were more… It was more “How’s* [partner]*?” “How’s the baby?” And then questions about her nausea and things like that. So, it didn’t feel that bad not being there.´* (Interview 2)

A perception emerged that midwives handled restrictions in different ways, with some midwives making individual adaptations and others not. Digital adjustments were made in some places, which enabled the inclusion of the partner during antenatal care appointments and ultrasound examinations. However, some were not allowed to use their phones to film or make video calls. The variety in terms of how maternity care handled digital adjustments affected whether parental education classes were offered; some were in digital form, and some were simply cancelled. Many participants expressed that digitalisation could have helped them to feel more included in the pregnancy and maternity care.

*´I am quite disappointed that they were so slow to offer digital alternatives. Because it would not have required much for an ultrasound, for example, to set up equipment and to be able to call, or my wife could have called with her cellphone and filmed for two minutes. It felt like I was very shut out of everything.´* (Interview 6)

**Reflecting upon the effects of disconnection.** The expectations to take an active part in maternity care were somewhat shattered due to restrictions on partners attending maternity care, and some participants described a feeling of loss of involvement in parenthood. The need for support from maternity care during pregnancy and in the postnatal ward was repeatedly described. Among interviewed partners,’ first-time parents in particular were in need of support, and some felt a sense of lack of preparation because of the cancelled parental education classes. However, some felt that they were well prepared; support from midwives made them feel reassured and confident as regards their life event of becoming parents.

*‘It feels like there are people who work full-time preparing first-time parents. And maybe those courses we missed are part of that. I felt, at least, that we were quite poorly prepared.’* (Interview 2)

Further, they identified the lack of participation in antenatal care and presence in the postnatal ward as a reason for struggling with the transition to parenthood. Feelings of exclusion were felt to create a sense of distance from and difficulty in comprehending the pregnancy, which affected the bonding with the unborn child.

*´Unlike the first child, when I was present during all antenatal care appointments and went to the parental education classes and we did everything together. Then I really felt like a parent. This time I only became a parent when he was born.´* (Interview 1)

Being separated from their partner and infant in the postnatal ward was described by the participants as having a negative impact on their experience, and a deferred connection with the infant was described. The participants needed time to recoup the connection with their child after birth. For some, this affected the way they planned for parental leave.

*´You definitely fall behind. That made us choose to do something different with the parental leave. It has had such a big impact… We changed it so that I would be home early to try to form a connection then, and it has actually worked quite well… but for the first four, five months, I was a father in name only. When I was alone with her, then it happened that we got a connection that is worth the name. So it was… that we changed the parental leave, which was our way out of me being a second-class parent.´* (Interview 10)

#### Striving to provide support

Participants repeatedly stated that they felt they had an important role to provide a sense of safety and support to their partner. They described forming a team together with their pregnant partner, this process was enhanced during antenatal care, and being together amplified feelings of team spirit and strengthened their relationship. They also desired to be together if they were to receive bad news regarding the pregnancy. During labour and in the postnatal ward, participants experienced that the healthcare professionals could not replace their presence. They worried that their partner would not receive adequate support from healthcare professionals in their absence due to crowded wards and lack of time. Some participants who experienced exclusion expressed that there was no other option than for them to be in the labour room with their partner in order to provide the support they needed.

*´I knew that* [my partner] *was having a bit of a rough time in* [the labour assessment room] *and that maybe there was a bit too much to do, that the staff were not coming that often to check… and then it would be good if I was there… it felt a bit like “let me in, I have to get in here”.´* (Interview 11)

The postnatal ward stay was frequently described as a challenging situation for the parent who gave birth in terms of being there alone with their infant. Among interviewed partners, first-time parents in particular expressed wanting to be there to share the burden. Tough decisions were made, and some couples even shortened their stay due to the family not being allowed to be together.

*´If I had been allowed in the postnatal ward, I’m pretty sure we would have stayed until it felt better. I think so. It was a lot of guessing when coming home using… YouTube and stuff like that.* (Interview 13)

Some participants who had had the opportunity to work from home acknowledged that the restrictions had some positive effects, and that their involvement in family life and feeling of togetherness with their partner increased during the pandemic.

*´I was at home the whole time. I didn’t have any, like, need to rush home in a car and pick her up* [for the birth]*. Instead, I was at home. So that aspect has only been positive.´* (Interview 15)

### Dealing with powerlessness

The second theme identified was *Dealing with powerlessness*. The participants described their daily lives as being clouded by a constant worry regarding the spread of the infection. However, they felt that they had to suppress these worries and navigate the dangers as best they could due to the inescapability of the pandemic. The participants described changing their ways of living, as well as missing out on certain important aspects of parenthood. This led to a mix of feelings, from a respectful attitude to the healthcare system and the need to put restrictions in place, to not being able to fully understand the arguments. In the end, the participants felt they simply had to accept and adapt to the situation.

#### A constantly present worrying mind

A persistent fear was described by the participants, who worried about infection of both themselves and their partner, which affected their daily lives to a great extent. There was a fear of how the COVID-19 virus would affect their pregnant partner and the foetus, since there was initially relatively little information available.

*´Our child was born before they started vaccinating pregnant women. We had read that there was a risk if you were infected that you could go into premature labour… that it would be a premature birth. That was kind of the information we had been given, so we were very, very careful. And I would say that we considered ourselves to be in an at-risk group and met very few people.´* (Interview 2)

The participants also feared they would miss the birth if they had symptoms of the virus when their partner went into labour, since support persons were not allowed into the labour ward if they had any COVID-19 symptoms. Most of the participants expressed that they had a backup plan of a support person who would be with the woman during labour. However, the participants described the thought of not being there for their partner when they gave birth as causing fear.

*´The stress that I might not be allowed to be present at birth was huge, and I think inhumane to put someone through. If my nose was the tiniest bit stuffed, then I wouldn’t be allowed at the birth.´* (Interview 3)

Many participants expressed a sense of powerlessness with regard to the risk for infection. Some of the participants were not able to work from home, which was stressful since they had no control over their social contacts and therefore could not minimise the risk of being infected. They also felt that it was hard to control this worry since whether they would be infected was out of their control. Many participants described how this worry, fear, and stress affected them negatively, and followed them from the start of the pregnancy until their partner had given birth.

*‘I would almost say that I was sick with worry during the spring […] I was… constantly checking… "Am I sick? Will something happen that will prevent me from being able to help and prevent me from being able to be part of what’s going on?” And it remained until we walked through the doors of the labour ward.’* (Interview 7)

Some of the worry eased as more knowledge about how the virus affected pregnant women and infants developed over time. Some participants felt relieved and less worried due to infants’ lower risk of illness as compared to adults.

*‘We understood that children were not considered a high-risk group, so we felt quite calm.’* (Interview 3)

#### Alternating between accepting and struggling with incomprehensible restrictions

The participants described respecting that restrictions were needed in healthcare. Most of the participants experienced the societal restrictions put in place as somewhat normal in their everyday lives, and expressed an acceptance of the situation. By focusing on the parts of pregnancy and labour that they could have an impact on, the participants found ways to cope with the limitations of the pandemic.

*‘It was a bit boring… not being allowed to participate* [during ultrasound]. *But at the same time, there and then, there wasn’t much to do, you just had to accept it as it was. But of course, if I had had the choice, I would have been there instead.’* (Interview 14)

At the same time the majority expressed frustration with their exclusion from other parts of maternity care, since they did not always feel that the restrictions were consistent or motivated. This made some of the participants question the restrictions that separated them from their family, as having one or a few hours with their newborn infant and then having to leave felt wrong.

*´So right after birth, I was kicked out, which is also strange from my side. Like, I just had a child, and suddenly I was sleeping at home, alone. It was a strange feeling.´* (Interview 5)

Some had friends who had been allowed to stay in the postnatal ward despite the restrictions, and others felt that it made no sense that they were allowed in the labour room but not the postnatal ward. Many participants had a hard time understanding the restrictions since they differed and changed a great deal during the pandemic.

*´They tested my partner for COVID-19 when we got to the labour ward, but not me. I was healthy, I didn’t have a fever, so I was allowed in the labour room. But when my partner was moved to the postnatal ward, I wasn’t allowed even though I wasn’t infected. Why couldn’t I come when I had already met all the personnel at the labour ward? Why did they only test my partner but not me?´* (Interview 9)

#### Adjusting life to minimise the risk of infection

The participants adapted their daily lives to the restrictions and to minimise the risk of infection. The participants described isolating for at least one week before the expected birth date in order to try to guarantee that they would be symptom-free and able to participate in the birth. The participants variously worked from home, took vacation days, and used parental leave to achieve this.

*´I didn’t meet anyone for ten days before the* [caesarean section] *surgery… If I hadn’t had the break then I would have tried… I would have made sure that I was free the week before in any case… I had some parental leave left from our older child; I probably would have been forced to use some of that.´* (Interview 10)

The majority of participants felt that it was relatively simple to adapt to the restrictions since everyone else in society had done the same. The participants described following the general restrictions in society, adjusting their social lives to meet people outside and maintaining a small network of friends and family that they had more regular contact with. Some participants expressed that the most negative restriction was the limited social possibilities. It was felt that social contact improves one’s mental wellbeing, and so the reduction in this had a negative impact on both the participants and their partners.

*´There is a great value that she as an expectant mother is feeling well before the birth, that she gets to move around and be active versus the still very small risk, but still the risk, of getting COVID… So it was… feelings of guilt, I don’t know, but it was a dilemma and a conversation we had to have and decide what was more worthwhile.´* (Interview 8)

## Discussion

This interview study explored partners’ experiences of pregnancy, labour, and postnatal care in relation to the COVID-19 pandemic in Sweden. The key findings were that partners had an expectation of involvement in maternity care, and that the maternity care restrictions greatly impacted the experience of pregnancy and early parenthood. Despite questioning the reasons for some of the restrictions, the participants adapted to the new situation.

Our findings show that inclusion in maternity care is seen as an important aspect of engaging the non-pregnant parent during pregnancy and labour. Participants wanted to be considered as natural parts of the support system for their partner during pregnancy, labour, and the beginning of parenthood, and felt irreplaceable as support persons for their partner. Similar to the findings of other studies [29, 30], the participants of this study valued being involved during pregnancy, labour, and postnatal care. Partners’ support for the pregnant woman and involvement in pregnancy, birth, and the early postpartum period are seen as beneficial for the whole family [31]. In the present study, becoming a parent was seen as an important and shared experience, and participation in maternity care was highly valued. Some participants felt that the exclusion that they experienced caused a feeling of distance from the pregnancy, and consequently a loss of bonding with their unborn infant. Not being included in maternity care was felt to lead to a deferred transition to parenthood. The feeling of ‘missing out’ have previously been reported by partners during the COVID-19 pandemic [13, 15, 16]. Other studies have shown a correlation between attending maternity care appointments and paternal engagement [32]. Some of the participants described the birth as the moment at which they started to feel like a parent, which was also found by Longworth and Kingdon, where fathers reported that fatherhood emerged at the time at which the infant was born [33]. It can be debated whether partner involvement in maternity care is as important for the transition to parenthood for second-time parents as it is for first-time parents, given that they already have parenting experience. Some second-time parents expressed that they were happy to have their first child before the pandemic. They compared their experiences with having their first child and concluded that involvement in maternity care enhances the transition to parenthood.

The feeling of exclusion in maternity care was also described by partners before the COVID-19 pandemic. A metasynthesis from high-resource settings reported that many fathers have a strong desire to support their partner, but that the maternity care system is often organised in a way that generates feelings of uncertainty, exclusion, and fear [34]. A longing for support from the healthcare system was expressed by the participants in our study. Swedish fathers have previously reported dissatisfaction with support from maternity care, and experiences of neglect [30, 35]. Other Swedish studies have reported that fathers feel symbolic but not practically involved in healthcare and request greater inclusion and support [36]. First-time fathers in particular expect antenatal care to extend beyond medical check-ups of mother and foetus, and could therefore be especially vulnerable to loss of support [37]. When providing support, healthcare professionals can play an important role in partners’ transitions to fatherhood, which can also help to strengthen the relationship between the father and the expectant mother [35].

The partners interviewed in the present study described a constant sense of worry. As found in other studies, partners were worried partly by the risk of severe disease, but more so by the possibility of missing the birth of their children [3]. Similar feelings were expressed by women who were pregnant during the COVID-19 pandemic [6]. In line with another Swedish study of expectant fathers’ experiences during COVID-19 [12], a great fear of missing the birth emerged in the results. Participants describing fearing that they would not be able to be there for their partner when she needed support during labour and postnatal care. Alternating between accepting and struggling with incomprehensible restrictions was reported. Participants expressed respecting the need for restrictions while also trying to cope, since the restrictions were not always consistent and the reasons behind not clear. They also described differences in terms of how the restrictions were put in place between midwives and maternity units. A systematic review of parental experiences of maternity care during the pandemic also shows that inconsistency in following restrictions was high across healthcare units, and the information provided was often felt to be confusing and unclear [16].

The partners experienced a need to adapt their everyday lives to minimise the risk of getting infected, and described changing their ways of living, for example in terms of how they socialised with friends and family. This led to a sense of missing out on social contact, as the participants expressed that socialising with other people was beneficial for their mental wellbeing. The COVID-19 pandemic highlighted the vital importance of social connection and vulnerability of many people to isolation and loneliness, which increased due to the restrictions [38]. The participants of this study described certain measures that would have improved their situations during the pandemic, and that could help the healthcare sector and other authorities when preparing for future pandemics: Digital meetings can secure partner involvement, engagement, and support without increasing the risk of infection; clearly stated and well-motivated restrictions would be easier to accept; and, where society values and expects partner involvement, financial compensation and/or the ability to work from home in the period immediately before the birth would alleviate the risk of partners missing the birth due to infection, and fear of this.

When interpreting the results of this study, the Swedish context needs to be taken into consideration. Firstly, Sweden is among the countries with the lowest maternal and neonatal mortality. While there were reports of increased risk of preterm birth in Sars-CoV2 infected women and severe COVID-19 in pregnant women, especially in the presence of further risk factors [39], very few cases of maternal mortality related to COVID-19 have been reported in Sweden. Thus, partner experiences in countries with considerably higher COVID19-related maternal mortality [40] will likely differ markedly from those presented in this study. Secondly, maternity care is offered free of charge, mainly through public care, and even private caregivers adhere strongly to regional and national guidelines. This means that there was no ability to pay for advanced private care, or to elude restrictions. Thirdly, Swedish society is regarded as one of the most equal societies in the world as regards gender issues, with a majority of non-birthing parents taking at least some parental leave [41]. The non-birthing parent generally attends the birth, and most partners of at least first-time mothers stay with their partner and newborn in the postnatal ward. In addition, Swedish maternity care is generally focused on including the partner, which likely affected the findings. Swedish partners’ expectations regarding antenatal and labour care will likely differ from those of partners in other contexts and transferability may therefore be low.

We aimed to increase the transferability of the study by including participants with a variation of ages and from diverse geographical regions in Sweden. Purposive sampling was used to recruit partners of women who gave birth during the COVID-19 pandemic. Although a diverse sample of both men and women of different ages and cultural backgrounds was recruited, a skew was found halfway through the data-collection process regarding education level and area of living. To compensate for this, the authors actively recruited participants with a lower educational level in another area of Sweden. The participants of this study were recruited from the northern and southwestern areas of Sweden to increase transferability. Wells et al. previously conducted a study on Swedish fathers’ experiences of becoming a father during the pandemic [12]. Some of the experiences in Wells et al.’s article are even found in our material, strengthening the findings in both articles, and implying a strong transferability. However, we may have reached a broader and more diverse population of partners, and our findings therefore provide an additional perspective on the area under study.

To create trustworthiness and increase credibility, we provided a detailed, step-by-step description of our data collection and analysis process. The interview guide was not pilot-tested, as it only included one broad question about the experience of having a child during the COVID-19 pandemic in relation to the domains of pregnancy, labour, and the postnatal period. Following the first interview, there was no identified need to revise the guide and the first interview was included in the results. Data was double-coded, and the analysis was constantly checked against the text and the aim of the study. An example of the analysis process is presented in Table 3 to enhance transparency. Several researchers confirmed the analysis and reflexivity through discussion of perspectives was practised, to mitigate potential biases. Through discussions within the research team, objectivity could be enhanced as individual preunderstandings were challenged. We aimed to include a rich data description with quotations supporting our results. The variation of experiences in the rich data sample increased the trustworthiness of the findings.

A possible limitation is that the present study focused on a two-year period, so we cannot ignore the possibility that the participants’ experiences differed due to changes in restrictions, access to vaccination, and growing knowledge during the pandemic. However, this may in fact strengthen the results, as a great variation in terms of experiences was achieved. Some of the children of the participants in this study were born at the end of 2021 or the beginning of 2022, and it can be discussed whether these participants were subject to restrictions in maternity care. Even though the restrictions were gradually eased toward the end of the data collection period with most restrictions lifted in the society in February 2022, the participants’ experiences of restrictions still varied. We also acknowledge the possibility of recall bias as some interviews were conducted up to two years postpartum. Moreover, four of the authors moderated the interviews, which possibly decreased the moderator bias of the data and strengthened the dependability. The research team consists solely of females, while the participants were mainly men. It is important to recognise that this may have limited our understanding of what the partners were going through.

## Conclusion

Partners of pregnant women who gave birth during the COVID-19 pandemic in Sweden were substantially affected by the restrictions put in place in order to reduce spread of the virus. They had a strong desire to be involved in maternity care and, when they were excluded, negative consequences regarding the transition to parenthood and support of their partner were perceived. Adjusting life to minimise the risk of infection and following restrictions that did not always appear logic was experienced as stressful. The results highlight that partners expect more than a safe pregnancy and birth from Swedish maternity care; partners wish to be involved in pregnancy and birth, and to receive clear information as part of their preparation for parenthood, and society and maternity care must decide how to address these needs. Some measures that may counteract the negative consequences of future pandemics are digital visits, engagement with and support for partners in maternity care, clear motivation for restrictions, and financial compensation or the ability to work from home before birth.
